# Menopausal Status and Disease Outcomes in Women With Rheumatoid Arthritis Receiving Anti-tumor Necrosis Factor (TNF) and Anti-IL-6 Therapies

**DOI:** 10.7759/cureus.110543

**Published:** 2026-06-09

**Authors:** Ghizlane Ghoullam, Imane El Binoune, Bouchra Amine, Ikram El Moubarik, Salma Zemrani, Samira Rostom, Rachid Bahiri

**Affiliations:** 1 Rheumatology, Ibn Sina Hospital Center, Faculty of Medicine and Pharmacy, Mohammed V University, Rabat, MAR

**Keywords:** biologic therapy, disease activity, estrogens, menopause, remission, rheumatoid arthritis

## Abstract

Introduction: Rheumatoid arthritis (RA) is a chronic inflammatory disease in which biologic therapies target remission or low disease activity. Sex hormones, particularly estrogens, influence immune responses, and hormonal changes across life stages may impact RA course and therapeutic outcomes. This study aimed to compare remission between menopausal and non-menopausal women with RA, assess time to first remission, and identify predictors of sustained remission.

Methods: A retrospective longitudinal study was conducted at the Rheumatology Department A of Ibn Sina Hospital Center, Faculty of Medicine and Pharmacy, Mohammed V University, Rabat-Salé, Morocco, including RA patients aged ≥18 years, treated with anti-TNF (tumor necrosis factor) or anti-IL-6 (interleukin-6) for ≥6 months, as monotherapy or combined with csDMARDS. Menopausal status was defined according to the World Health Organization (WHO) criteria. Patients were classified into menopausal (M-Gr) and non-menopausal (NM-Gr) groups. DAS28 and SDAI assessed disease activity. Remission was defined as DAS28-CRP ≤2.6 and sustained remission as ≥6 months.

Results: Seventy-five patients were included: 49 (65.3%) in the menopausal group and 26 (34.7%) in the non-menopausal group. The mean age was 55 ± 13 years, and the mean disease duration was 15 ± 8 years. Anti-TNFα therapy was used in 55 (73.3%) patients and anti-IL-6 therapy in 20 (26.7%) patients. In addition, 50 (66.7%) patients had prior exposure to bDMARDs. Remission was achieved in 25 (51.1%) patients in the M-Gr and 12 (46.2%) in the NM-Gr (p = 0.68). The median time to first remission was 12 weeks in the M-Gr compared with 14 weeks in the NM-Gr, with no statistically significant difference (p = 0.08). Sustained remission was observed in 23 (46.9%) patients in the M-Gr and eight (30.7%) in the NM-Gr (p = 0.17). Higher baseline swollen joint count (OR = 0.28, p = 0.02) and higher baseline tender joint count (OR = 0.35, p = 0.03) were independently associated with lower odds of sustained remission. Furthermore, Menopausal status was not independently associated with sustained remission (OR = 0.19, p = 0.43).

Discussion: Our study indicates that menopausal status does not significantly influence remission rates in women with RA receiving anti-TNF or anti-IL6 therapy. These findings suggest that biologic treatments may overcome hormonal-related differences in inflammatory activity.

Conclusion: Menopausal status was not significantly associated with remission outcomes in RA patients treated with biologic therapy.

## Introduction

Rheumatoid arthritis (RA) is a chronic autoimmune disease marked by persistent joint inflammation and systemic involvement and has destructive potential, leading to significant morbidity and disability if left untreated. RA affects approximately 0.5-1% of the adult population worldwide and disproportionately affects women, with a female-to-male ratio of about 3:1. Moreover, sex-related differences have been reported in disease expression, with women generally experiencing higher disease activity, greater symptom burden, and worse functional outcomes compared with men [1-2].

Hormonal factors are thought to play a central role in these sex-related differences. Sex hormones, particularly estrogen and progesterone, are key regulators of immune responses. Their fluctuations during puberty, pregnancy, postpartum, and menopause have been shown to influence RA onset and progression [3]. Studies suggest that estrogen has immunomodulatory effects, potentially explaining the lower incidence of RA in premenopausal women and the disease's exacerbation after menopause [4]. Estrogen deficiency has been linked to elevated levels of pro-inflammatory cytokines, such as tumor necrosis factor-alpha (TNF-α) and interleukin-6 (IL-6), which play a pivotal role in RA pathogenesis. These hormonal changes may also influence response to biologic therapies, highlighting an area of growing clinical interest [5].

Over the past two decades, biologic therapies targeting key inflammatory pathways, particularly TNF-α and IL-6, have profoundly transformed RA management. Current treatment strategies aim to achieve remission or low disease activity, thereby preventing structural damage and preserving quality of life [1]. However, therapeutic response to biologic agents remains heterogeneous and may be influenced by patient-related factors, including hormonal status.

While several studies have explored the association between menopausal status and disease severity, functional impairment, or radiographic progression in RA, data regarding the influence of menopause on treatment response are limited. Importantly, remission represents the primary therapeutic target in contemporary RA management, yet the impact of menopausal status on remission achievement under biologic therapy remains insufficiently investigated.

Given these gaps, the present study aimed to investigate the association between menopausal status and remission outcomes in women with RA receiving biologic therapy. The primary objective was to compare remission rates at six months after initiation of the current biologic therapy between menopausal and non-menopausal women with RA. Secondary objectives included comparing the time to first remission, rates of sustained remission (≥6 months), and identifying factors associated with sustained remission in both groups. This study was exploratory in nature and aimed to generate hypotheses regarding the impact of menopausal status on treatment response in RA.

## Materials and methods

Study design

This was a retrospective observational study with longitudinal follow-up, including adult women (>18 years) diagnosed with RA according to the 2010 American College of Rheumatology/European Alliance of Associations for Rheumatology (ACR/EULAR) classification criteria [6], conducted at the Rheumatology Department A of Ibn Sina Hospital Center, Faculty of Medicine and Pharmacy, Mohammed V University, Rabat-Salé, Morocco.

Participants were receiving biologic therapy, either as monotherapy or in combination with conventional synthetic DMARDs (csDMARDS). Preliminary results from this study were previously presented as a poster at the EULAR 2025 Annual Meeting. The study protocol received approval from both the local institutional review board and the national ethics committee: the Ethics Committee for Biomedical Research at Mohammed V University, Faculty of Medicine and Pharmacy of Rabat (CERB140-25). The study was conducted in accordance with the principles of the Declaration of Helsinki for research involving human subjects, and written informed consent was obtained from all participants.

Patient selection

A total of 95 RA patients were initially screened for eligibility. Twenty patients were excluded for the following reasons: biologic therapy duration less than six months (n = 12) and missing data, including inflammatory markers, joint counts, or DAS28 components (n = 8). The final study population included 75 patients, stratified according to their menopausal status based on the World Health Organization (WHO) criteria (absence of menstruation for ≥12 months without pathological cause) into menopausal (M-Gr, n = 49) and non-menopausal (NM-Gr, n = 26) groups. This number represents all patients in our center who are on biologic treatment and are regularly followed up with, ensuring complete and reliable data. Only female patients were included to specifically investigate and categorize the characteristics and outcomes of this subpopulation, with a focus on the impact of menopausal status. Future studies may expand to include male patients to allow comparative analyses between sexes.

Outcome definitions

Menopausal status was defined according to the WHO criteria as the absence of menstruation for 12 consecutive months after excluding other causes [7,8]. Disease activity was assessed using DAS28-ESR and DAS28-CRP, with remission defined as DAS28-CRP ≤2.6, low disease activity as DAS28-CRP >2.6 and ≤3.2, and the time to first remission defined as the interval between the initiation of biologic therapy and the first documented remission among patients who achieved remission during follow-up [9]. The DAS28 is a validated composite disease activity score widely used in RA research and does not require specific licensing for academic use.

Data collection and assessment

Data were collected from patient records at the initiation of the current biologic therapy and during subsequent follow-up visits (weekly or monthly) to allow longitudinal observation. Collected variables included clinical data (age at inclusion, age at menopause, duration since menopause, body mass index (BMI), time since diagnosis, disease duration, onset pattern, serological status, Health Assessment Questionnaire (HAQ) score originally developed by Fries et al. (1980) [10], and comorbidities related to RA, such as sicca syndrome, osteoporosis, and organ involvement (renal, pulmonary, and cardiac)), biological data (inflammatory markers, such as erythrocyte sedimentation rate (ESR) and C-reactive protein (CRP)), and therapeutic data (the current biologic therapy, conventional synthetic DMARDs, and corticosteroid use). Patients were followed at regular intervals (approximately every two to four weeks) according to routine clinical practice in a rheumatology day-care setting.

Statistical analysis

Data analysis was performed using JAMOVI version 2.3.28. Continuous variables were expressed as mean ± standard deviation (SD) or median with interquartile range, depending on their distribution. Categorical variables were summarized as frequencies and percentages. Comparisons between groups were conducted using Student’s t-test for normally distributed continuous variables or the Mann-Whitney U test for non-normally distributed variables. Categorical variables were analyzed using the Chi-square test or Fisher’s exact test, as appropriate. The time to first remission was analyzed as a continuous variable and compared between groups using the Mann-Whitney U test. 

Multivariate analysis was carried out using a logistic regression model to identify factors associated with sustained remission. A p-value < 0.05 was considered statistically significant in all analyses.

## Results

A total of 75 RA patients were included, with a mean age of 55 ± 13 years and a mean disease duration of 15 ± 8 years. Among them, 55 (73.3%) were treated with anti-TNFα and 20 (26.7%) with anti-IL6. Prior exposure to bDMARDs was reported in 50 (66.7%) patients, while 25 (33.3%) were biologic-naïve. Twenty-four (32%) patients presented with a difficult-to-treat (D2T) form of RA.

At baseline, the menopausal group (M-Gr, n = 49) was significantly older and had a longer disease duration and diagnostic delay compared with the non-menopausal group (NM-Gr, n = 26). In addition, higher rates of hypertension, diabetes, and osteoporosis were observed in the menopausal group. However, baseline disease activity scores (DAS28-CRP, DAS28-ESR, and SDAI), inflammatory markers, pain intensity, functional status, joint counts, immunological characteristics, and most treatment-related variables did not differ significantly between the two groups (Table 1). 

**Table 1:** Characteristics of our population stratified by menopausal status (at the initiation of the current biologic therapy) (*): Expressed as mean and standard deviation or median and interquartile range (IQR). (**): Expressed as numbers and percentages. BMI: body mass index; protein, HAQ score: Health Assessment Questionnaire score, anti-CCP: anti-cyclic citrullinated peptide antibodies, DAS28: Disease Activity Score for 28 joints, CRP: C-reactive protein, ESR: erythrocyte sedimentation, VAS: Visual Analog Scale, bDMARD: biologic disease-modifying anti-rheumatic drugs, csDMARDs: conventional synthetic disease-modifying antirheumatic drugs, SDAI: Simplified Disease Activity Index, D2T: difficult to treat Student’s t-test (t) was used for normally distributed variables, Mann–Whitney U test (U) for nonnormally distributed variables, and chi-square test (χ²) for categorical variables.

Variable	Menopausal (M-Gr, n = 49)	Non-menopausal (NM-Gr, n = 26)	Test statistic	p-value
Age (years)*	62.9±7.8	39.2±7.4	t = -12.8	<0.001
BMI, kg/m2 *	27 (23.2-32)	25 (23.4-29.3)	U = 550	0.33
Hypertension**	20 (40.8%)	1 (3.8%)	χ² = 14.8	<0.001
Diabetes**	16 (32.7%)	0 (0%)	χ² = 10.8	0.001
Disease duration, years*	16.9±7.7	12.6±7.4	t = -2.33	0.02
Diagnostic delay, years *	3 (1-4)	1 (1-3)	U = 432	0.01
Immuno-positivity**	37 (75.6%)	18 (69.3%)	χ² = 0.34	0.55
VAS pain*	6.33±1.2	6±1.02	t = -1.18	0.22
Tender joint count (TJC)*	7 (6-11)	6.5 (4-12)	U = 597	0.65
Swollen joint count (SJC)*	6 (4-9)	5.5 (4-9.5)	U = 625	0.89
CRP (mg/l)*	14 (7-28)	15.8 (8.5-25.5)	U = 578	0.51
ESR (mm/h)*	44.3±23.4	37.6±22.5	t = -1.19	0.22
DAS28-CRP*	4.9±0.9	5.1±1.1	t = 0.62	0.53
DAS28-ESR*	5.4±0.9	5.2±1.02	t = -0.66	0.51
SDAI*	28.7±8.9	31.7±15.7	t = 1.08	0.28
HAQ score *	1.21±0.7	0.9±0.4	t = -1.79	0.12
Biologic therapy (anti-TNF / anti-IL-6)**	anti-TNF : 33 (67.4%) anti-IL-6 : 16 (32.6%)	anti-TNF : 22 (84.6%) anti-IL-6 :4 (15.4%)	χ² = 7.06	0.11
Prior bDMARD exposure**	31 (63.3%)	19 (73.2%)	χ² = 1.08	0.39
difficult-to-treat (D2T) disease**	17 (34.7%)	7 (26.9%)	χ² = 0.47	0.49
csDMARDs, use**	16 (32.7%)	10 (38.5%)	χ² = 0.73	0.69
Coxitis**	9 (18.4%)	6 (23.1%)	χ² = 0.23	0.62
C1-C2 subluxation**	1 (2.2%)	3 (11.6%)	χ² = 3.03	0.08
Osteoporosis**	31 (63.3%)	8 (30.8%)	χ² = 7.19	0.007

Among postmenopausal patients (n = 49), the mean age at menopause was 50.4 ± 4.6 years, and the mean duration since menopause was 12.5 ± 6.9 years.

Overall, remission at six months was observed in 25 (51.1%) of 49 menopausal patients and 12 (46.2%) of 26 non-menopausal patients, with no statistically significant difference between the groups (p = 0.68).

The median time to first remission was 12 weeks in menopausal patients versus 14 weeks in non-menopausal patients (p = 0.08). Persistent remission for ≥6 months was observed in 23 (46.9%) menopausal patients compared to eight (30.7%) non-menopausal patients (p = 0.17) (Table 2).

**Table 2:** Remission outcomes by menopausal status (*): Expressed as mean and standard deviation or median and interquartile range (IQR). (**): Expressed as numbers and percentages. DAS28: Disease Activity Score for 28 joints, CRP: C-reactive protein, ESR: erythrocyte sedimentation rate, VAS: Visual Analog Scale

Variable	Menopausal (M-Gr, n = 49)	Non-menopausal (NM-Gr, n = 26)	Test statistic	p-value
Rates of remission at six months **	25 (51.1%)	12 (46.2%)	χ² = 0.17	0.68
Time to first remission, weeks*	12 (8–17)	14 (8–29)	U = 428	0.08
Rates of persistent remission ≥6 months**	23 (46.9%)	8 (30.7%)	χ² = 1.83	0.17
DAS28-CRP at remission *	2.4 (1.7-2.83)	2.75 (1.94-2.88)	U = 527	0.22
DAS28-ESR at remission *	2.33 ± 0.41	2.35 ± 0.55	t = 0.19	0.35
Tender joint count (TJC) at remission *	0 (0-2)	1 (0-2)	U = 448	0.63
Swollen joint count (SJC) at remission *	0 (0-2)	0 (0-1)	U = 476	0.95
CRP at remission *	1.51 (0.97-4.28)	3.85 (1-6.13)	U = 376	0.15
ESR at remission *	14 (6-19.3)	16.5 (7.25-25.3)	U=457	0.75
VAS pain at remission *	1 (0-2)	2 (0-2)	U = 444	0.57

In the multivariate logistic regression analysis, the model was adjusted for age, disease duration, baseline DAS28-CRP/DAS28-ESR, prior biologic exposure, corticosteroid use, difficult-to-treat disease status, and menopausal status (main exposure variable), as well as menopause-related variables including age at menopause and duration since menopause.

Higher baseline swollen joint count (OR = 0.28, p = 0.02) and higher baseline tender joint count (OR = 0.35, p = 0.03) were independently associated with lower odds of sustained remission. In contrast, menopausal status did not remain significantly associated with sustained remission in the adjusted model (OR = 0.19, p = 0.43). Neither age at menopause nor duration since menopause showed a significant association with sustained remission. No significant associations were observed for other covariates, including age, disease duration, baseline DAS28-CRP, DAS28-ESR, prior biologic exposure, D2T status, and corticosteroid use (Table 3).

**Table 3:** Factors predicting persistent remission in the overall study population (*): DAS28: Disease Activity Score for 28 joints, CRP: C-reactive protein, ESR: erythrocyte sedimentation rate, CS: corticosteroid, SDAI: Simplified Disease Activity Index, D2T: difficult to treat

	OR	IC95% lower-upper	P-value
Age	1.04	0.97-1.12	0.27
Disease duration, years	0.95	0.81-1.12	0.55
Diagnostic delay, years	1.21	0.86-1.48	0.37
Swollen joint count (SJC) at M0	0.28	0.09-0.84	0.02
Tender Joint Count (TJC) at M0	0.35	0.14-0.90	0.03
DAS28-CRP* at M0	2.54	0.56-11.43	0.22
DAS 28-ESR* at M0	0.56	0.09-3.55	0.54
ESR* at M0	1.01	0.95-1.07	0.76
CRP* at M0	0.91	0.82-1.02	0.10
D2T*	2.22	0.17-29.03	0.54
Fibromyalgia	1.19	0.37-3.79	0.76

## Discussion

RA is influenced by hormonal fluctuations throughout a woman’s life, particularly estrogen levels, which are known to modulate immune responses and inflammatory activity. The higher prevalence of autoimmune diseases, including RA, in women compared to men suggests a pivotal role for estrogen in their pathogenesis. Menopause, characterized by a substantial decline in estrogen levels, affects multiple physiological systems. Nevertheless, the precise effects of estrogen deficiency on immune function remain incompletely understood [11]. Data on the impact of menopause on therapeutic response to biologic treatments remain limited.

Estrogens play a complex, concentration- and receptor-dependent role in RA pathogenesis. ERα and ERβ are differentially expressed on immune cells, with ERα predominant on CD4+ T cells and macrophages, and ERβ higher on B cells, influencing cytokine production and immune responses. Physiological estradiol levels may stimulate pro-inflammatory cytokines, such as TNF-α, IL-1β, and IL-6, while higher concentrations promote anti-inflammatory cytokines (IL-4, IL-10, and TGF-β). Hydroxylated estrogen metabolites in synovial fluid can further modulate local immune activation. Therefore, estrogen has immunosuppressive and immunostimulatory effects depending on its level [12].

Menopause is associated with a marked decrease in circulating estrogens, which could theoretically reduce pro-inflammatory stimulation via cytokines such as TNF-α and IL-6 [13].

Our study examined the relationship between menopausal status and both remission rates and disease outcomes in women with RA receiving anti-TNF and anti-IL6 therapy. Overall remission was achieved in 51.1% of postmenopausal women compared with 46.2% of premenopausal women, and this difference did not reach statistical significance, suggesting that menopausal status alone does not substantially influence remission under biologic therapy. Median time to first remission and the proportion of patients achieving persistent remission were also comparable between menopausal and non-menopausal patients, suggesting that menopause alone does not substantially influence treatment outcomes under biologic therapy. These findings support the hypothesis that biologic therapies targeting TNF-α and IL-6 may override hormonal-related differences in inflammatory activity, leading to comparable remission outcomes regardless of menopausal status. While our study did not find a significant difference in remission outcomes between menopausal and non-menopausal women, previous meta-analytic data indicate that the risk of developing RA is greater in postmenopausal women than in their premenopausal counterparts (OR 1.35, 95% CI 1.04-1.67), and those experiencing early menopause have an even greater risk (OR 2.97, 95% CI 1.73-4.22) [14]. It is therefore important to differentiate factors influencing disease onset from those modulating treatment response [15,16].

In our study, we focused on pre- and postmenopausal women and observed comparable overall and persistent remission rates between the two groups under biologic therapy. Daraghmeh et al. investigated remission across premenopausal, perimenopausal, and postmenopausal women and reported that perimenopausal women were less likely to achieve remission, while postmenopausal women had intermediate outcomes and premenopausal women had the highest likelihood of remission [17].

Although the inclusion of perimenopausal women in their study highlighted a transitional period with hormonal fluctuations that may temporarily reduce remission likelihood, our findings indicate that when only pre- and postmenopausal women are considered, biologic therapy effectively reduces the impact of menopausal status on remission.

Although our study focused primarily on clinical remission and time to first remission, it is important to consider other disease outcomes such as functional status and structural progression.

Previous research indicates that postmenopausal women with RA often experience a more rapid decline in functional capacity, as measured by the Health Assessment Questionnaire (HAQ), compared to premenopausal women [18,19]. The partial loss of estrogen’s immunomodulatory effects contributes substantially to the negative disease outcomes in women with RA undergoing menopause [20].

Some studies suggest an association between menopausal status and accelerated radiographic progression. In a large observational cohort including 1,667 women with RA, postmenopausal women exhibited significantly higher baseline functional disability and erosion scores compared with premenopausal women. Over a median follow-up of more than five years, functional disability, assessed by the HAQ, progressed more unfavorably in postmenopausal women, particularly in those with an earlier age at menopause, even after adjustment for age, disease duration, disease activity, serological status, and treatment. By contrast, no significant difference in radiographic erosion progression was observed between pre- and postmenopausal women [21].

These findings suggested that even in the presence of similar remission rates under biologic therapy, postmenopausal women may still be at higher risk for long-term functional deterioration and joint damage.

Incorporating structural and functional outcomes in future studies would provide a more comprehensive understanding of the impact of menopause on the RA disease course.

We found that baseline clinical factors, including joint counts (SJC and TJC), were stronger predictors of sustained remission in both groups. Higher initial swollen joint count and tender joint count were negatively associated with sustained remission, indicating that greater baseline joint involvement may reduce the likelihood of achieving long-term disease control.

In line with these findings, Martire et al. analyzed 89 RA patients and identified several factors significantly associated with sustained remission, including lower baseline DAS28 and HAQ scores, negative CRP, and absence of erosions [22].

Their study utilized both DAS28 and the ACR-EULAR Boolean criteria to define sustained remission, highlighting the importance of combining objective and composite measures to assess long-term disease control. These results suggest that, beyond menopausal status, a combination of modifiable factors (such as disease activity and inflammatory markers) and non-modifiable factors (such as gender and structural joint damage) play a crucial role in achieving and maintaining remission.

In a large multinational cohort of over 6,000 RA patients treated with adalimumab, Burmester et al. demonstrated that younger age, lower baseline DAS28, lower CRP levels, and lower HAQ scores were significant predictors of achieving clinical remission or minimal disease activity [23].

Strengths and limitations

Our study has several strengths. First, it specifically focused on remission and persistent remission under biologic therapy, which are clinically relevant outcomes in RA. The evaluation of both clinical and functional parameters (HAQ, SDAI, and joint counts) allowed for a comprehensive assessment of factors influencing remission. In addition, all patients were managed in a specialized rheumatology center, ensuring standardized follow-up and treatment strategies.

However, several limitations must be acknowledged. The monocentric design may limit the generalizability of the findings. In addition, the relatively small sample size may reduce statistical power, particularly for subgroup analyses and persistent remission outcomes. The exclusion of patients with missing clinical and laboratory data may have introduced selection bias, although it ensured the completeness and reliability of outcome assessment. Time to first remission was analyzed descriptively among patients who achieved remission. As patients without remission were not included in this analysis, classical time-to-event approaches, such as Kaplan-Meier or Cox regression, were not applied. Age at menopause and time since menopause were available in the dataset and included in the analysis; however, surgical menopause status was not systematically recorded. In addition, no patient in our cohort had received hormone replacement therapy. Therefore, residual confounding related to unmeasured menopause-related factors cannot be excluded. Finally, given the limited sample size, the multivariable logistic regression model included a restricted number of clinically relevant covariates; nevertheless, the possibility of overfitting cannot be excluded, and results should therefore be interpreted with caution.

Overall, while these limitations should be considered, the strengths of our study lie in its focus on clinically meaningful outcomes and its contribution to the limited literature exploring the role of menopausal status in RA remission under biologic therapy.

## Conclusions

Menopausal status may influence disease course in RA; however, in this retrospective cohort, no significant association was observed between menopausal status and remission rates under biologic therapy. Baseline disease characteristics remained the main predictors of sustained remission. These findings highlight the importance of early disease control regardless of menopausal status. Furthermore, larger prospective studies are needed to confirm these results.
